# Long-term follow-up of givosiran treatment in patients with acute intermittent porphyria from a phase 1/2, 48-month open-label extension study

**DOI:** 10.1186/s13023-024-03284-w

**Published:** 2024-10-03

**Authors:** Eliane Sardh, Manisha Balwani, David C. Rees, Karl E. Anderson, Gang Jia, Marianne T. Sweetser, Bruce Wang

**Affiliations:** 1 CMMS - Centre for Inherited Metabolic Diseases, Karolinska University Hospital, Karolinska Institutet, Solna, 171 64 Stockholm, Sweden; 2 Department of Genetics and Genomic Sciences, Icahn School of Medicine, New York, NY USA; 3 Comprehensive Cancer Centre, King’s College Hospital, London, UK; 4 Department of Internal Medicine, Division of Gastroenterology and Hepatology, University of Texas Medical Branch, Galveston, TX USA; 5 Medical Affairs Statistics, Alnylam Pharmaceuticals, Cambridge, MA USA; 6 Clinical Development, Alnylam Pharmaceuticals, Cambridge, MA USA; 7 Department of Medicine and UCSF Liver Center, University of California San Francisco, San Francisco, CA USA

**Keywords:** Acute hepatic porphyria (AHP), Acute intermittent porphyria (AIP), Givosiran, RNA interference, Delta-aminolevulinic acid (ALA), Porphobilinogen (PBG), Hemin

## Abstract

**Background:**

Acute hepatic porphyria is a group of multisystem disorders of which acute intermittent porphyria is the most common subtype. Givosiran, a subcutaneously administered RNA interference therapeutic targeting liver ALAS mRNA, is approved for treating these disorders. This Phase 1/2 open-label extension study (NCT02949830) evaluated the long-term safety and efficacy of givosiran in adults with acute intermittent porphyria, with follow-up of up to 48 months, which is the longest follow-up of givosiran treatment to date. Participants were adults aged 18–65 years who completed part C of the Phase 1 givosiran study (NCT2452372).

**Methods:**

Enrollees received givosiran for up to 48 months. Primary and secondary endpoints included the incidence of adverse events, changes in urinary delta-aminolevulinic acid (ALA) and porphobilinogen (PBG) levels, annualized rate of porphyria attacks, and annualized hemin use. Quality of life was assessed using the EQ-5D-5L instrument as an exploratory endpoint.

**Results:**

Sixteen patients (median age: 39.5 years) participated. Common adverse events included abdominal pain, nasopharyngitis, and nausea (50% each), with injection-site erythema (38%) and injection-site pruritus (25%) noted as frequent treatment-related reactions. Givosiran therapy reduced annualized rates of porphyria attacks and hemin use by 97% and 96%, respectively. From months > 33 to 48, all patients were free from attacks requiring significant medical intervention and did not use hemin. There were substantial reductions in median urinary ALA and PBG of 95% and 98%, respectively. Additionally, a clinically meaningful improvement in quality of life was observed.

**Conclusions:**

In the longest follow-up of givosiran-treated patients reported to date, the therapy maintained an acceptable safety profile and demonstrated sustained improvements in clinical outcomes over 4 years in patients with acute intermittent porphyria.

**Supplementary Information:**

The online version contains supplementary material available at 10.1186/s13023-024-03284-w.

## Background

Acute hepatic porphyria (AHP) encompasses a group of rare genetic metabolic progressive disorders, each manifesting with severe neurovisceral attacks that can be acute and debilitating [1–3]. Patients with AHP often develop chronic symptoms and long-term complications, emphasizing the need for proactive, continuous management [3]. AHP encompasses 4 porphyria subtypes: acute intermittent porphyria (AIP; the most common and symptomatic), variegate porphyria (VP), hereditary coproporphyria (HCP), and delta-aminolevulinic acid (ALA) dehydratase deficiency [3]. In each AHP subtype, a genetic defect that leads to a deficiency in one of the enzymes of liver heme biosynthesis causes depletion of the hepatic free heme pool and induction of the rate-controlling enzyme of the heme biosynthesis pathway, aminolevulinate synthase 1 (ALAS1) [4–7]. AHP is a variable condition, and manifestations can be multisystemic [1]. Both chronic and acute symptoms of AHP impact patient quality of life (QoL), contributing to substantial burden of disease [8]. Patients with AHP can present with nonspecific symptoms, which frequently results in misdiagnosis and inadequate management [5]. To better understand if treatments are working to alleviate symptoms over time, long-term follow-up of patients is important.

Chronic manifestations of AHP include pain, fatigue, and nausea [8]. Acute attacks are characterized by severe abdominal pain, nausea, vomiting, tachycardia, hypertension, hyponatremia, mental status changes, and muscle weakness [2, 9–15]. Patients with chronic pain may require long-term analgesia, including opioids [5, 16]. Prior to the approval of givosiran, management options for acute AHP attacks were limited to avoidance of attack triggers and administration of intravenous (IV) hemin. For patients experiencing recurrent attacks, prophylactic therapy with hemin has been used in clinical practice [17, 18]. However, the effects of hemin are short-lived, and therapeutic efficacy can decline with prolonged or repeated use [19]. Additionally, repeated and prophylactic hemin use confers the risk of adverse events (AEs) including venous damage and thrombophlebitis, coagulation abnormalities, and secondary iron overload [13, 17, 20].

As a consequence of ALAS1 induction in AHP, overproduction and accumulation of the neurotoxic heme intermediates ALA and porphobilinogen (PBG) occur, causing nervous system injury and damage to other organs, including the liver and kidneys [7, 16, 21]. Substantial elevation in urinary PBG, generally > 3 times the upper limit of normal (ULN) [1, 22], can establish a diagnosis of AHP. This threshold enables a high degree of diagnostic specificity, as PBG elevation of this magnitude does not result from any medical condition other than AIP, VP, or HCP [1, 22]. An acute porphyria attack is characterized by a significantly increased urinary PBG/creatinine ratio, typically > 10 times the ULN, or > 10 μmol/mmol creatinine if the ULN is ≤ 1 μmol/mmol creatinine (eg, when measured by mass spectrometry) [9].

Givosiran is a subcutaneously administered RNA interference therapeutic approved for treatment of AHP in adults (United States, Canada, Brazil) and adolescents age ≥ 12 years (European Economic Area, Switzerland, Japan) [23–28]. Givosiran lowers ALAS1 messenger RNA (mRNA) expression in the liver, thereby preventing accumulation of ALA and PBG [29–32]. Findings from a Phase 1 study of givosiran in patients with AIP (NCT02452372) (part C, *n* = 17 experiencing ≥ 2 attacks within 6 months before the run-in period or receiving scheduled hemin prophylaxis at the start of the run-in period) demonstrated that compared with placebo, once-monthly givosiran therapy led to a sustained decline in urinary ALA and PBG concentrations and a reduction in the annualized rate of porphyria attacks (defined as attacks leading to hospitalization, urgent health care visits, or use of IV hemin at home) and annualized days of hemin use [31]. Most AEs were mild to moderate in severity, with similar rates observed in the givosiran and placebo groups [31]. The Phase 3 ENVISION trial (NCT03338816) of givosiran versus placebo (*N* = 94), which included a 6-month double-blind period followed by a 30-month open-label extension (OLE), demonstrated safety and efficacy outcomes with givosiran that were consistent with those seen in the Phase 1 study. Compared with placebo, treatment with givosiran reduced the occurrence of porphyria attacks and hemin use, and lowered levels of ALA, PBG, and daily worst pain; these improvements were maintained in the OLE period [29, 33]. These results demonstrated that givosiran treatment of up to 36 months yielded consistent benefits. We wanted to evaluate if the improvements in clinical outcomes observed for AIP could be maintained even longer term, so we examined other long-term givosiran data that became available—namely, data from our Phase 1/2 OLE study in which patients were treated for up to 48 months.

The multicenter, Phase 1/2, OLE study (NCT02949830) was conducted to evaluate the long-term safety and efficacy of givosiran in patients with AIP who completed part C of the Phase 1 study (NCT02452372) [31]. Here we report results from the Phase 1/2 OLE study in patients with AIP receiving givosiran for up to 48 months—the longest follow-up of patients treated with givosiran reported to date.

## Methods

### Study design

This Phase 1/2 multicenter OLE study, conducted from May 2015 to November 2021 across 5 centers (3 in the United States and 2 in Europe), was designed to evaluate the long-term safety and clinical activity of givosiran in patients with AIP with ≥ 2 attacks within 6 months before the run-in period or receiving scheduled hemin prophylaxis at the start of the run-in period and who had completed part C of the prior Phase 1 study [31]. The study protocol, amendments, and informed consent form were reviewed and approved by an independent ethics committee or a site-specific institutional review board. The study was conducted in accordance with Good Clinical Practice guidelines and the provisions of the Declarations of Helsinki and Istanbul [34–36]. All patients provided written informed consent.

Patients in part C of the Phase 1 study were randomized (3:1) to one of two doses of givosiran (2.5 mg/kg or 5.0 mg/kg) or placebo once-monthly (total of 4 injections) or once every 3 months (total of 2 injections) during a 12-week treatment period and followed for an additional 12 weeks (approximately 6 months overall) [31]. At entry into the Phase 1/2 OLE study, patients received givosiran at 2.5 mg/kg once-monthly or 5.0 mg/kg once-monthly or every 3 months. After a review of the emerging safety, efficacy, and pharmacokinetic and pharmacodynamic modelling data from the Phase 1 study, all patients transitioned to once-monthly doses of 2.5 mg/kg starting in August 2017, and remained on this dose for the duration of the Phase 1/2 OLE study. Patients in the Phase 1/2 OLE study were treated with givosiran for up to 48 months.

### Patients

The study population consisted of patients aged 18 to 65 years diagnosed with AIP, confirmed by a pathogenic variant in the *hydroxymethylbilane synthase* (*HMBS*) gene, which codes for PBG deaminase. Eligible patients experienced recurrent porphyria attacks, defined as two or more attacks within the 6 months prior to the Phase 1 study run-in or receiving scheduled hemin prophylaxis at the start of the Phase 1 study run-in. Additionally, eligible patients had completed part C of the Phase 1 parent study [31]. To qualify for part C of the Phase 1 parent study, patients were either not on a scheduled prophylactic hemin therapy regimen or agreed to discontinue any scheduled hemin prophylaxis during the 4- to 24-week run-in and up to 12-week treatment period [31]. Patients were excluded if they had an alanine aminotransferase (ALT) concentration ≥ 2.0 × ULN, total bilirubin ≥ 2 mg/dL, or estimated glomerular filtration rate (eGFR) of ≤ 30 mL/min/1.73m^2^.

### Endpoints and assessments

The primary endpoint was the incidence of AEs; key secondary endpoints were changes in urine ALA and PBG levels and clinical activity of givosiran as assessed by the frequency and characteristics of porphyria attacks (defined as attacks leading to hospitalization, urgent health care visits, or use of IV hemin at home) and change in the number of hemin doses administered. Health-related QoL as assessed by changes in Euro Quality of Life Health State Profile Questionnaire (EQ-5D-5L) and EuroQoL visual analogue scale (EQ-VAS) scores were evaluated as an exploratory endpoint.

Safety assessments consisted of monitoring AEs, vital signs, results from physical examinations, electrocardiogram measurements, and clinical laboratory assessments. AEs were coded according to the Medical Dictionary for Regulatory Activities (MedDRA) Version 23.0.

Patients and caregivers were given a diary to record acute porphyria attacks throughout the study; pain assessments and narcotic use were recorded on a daily basis through Month 9. Patients were encouraged to report to the clinical study site if at any time between study entry and the end of study visit they experienced a porphyria attack. If they were unable to report to the clinical study site, they were to collect a urine sample using a home collection kit and send it to the clinical study site, if possible. A porphyria attack was defined as any event with the preferred term *porphyria* recorded on an adverse event electronic case report form. Composite porphyria attacks were defined as attacks requiring hospitalization, an urgent health care visit, or administration of IV hemin at home. Levels of ALA and PBG were evaluated in urine samples by a central laboratory; pre-dose samples were collected on dosing days. Additionally, changes in circulating hepatic ALAS1 mRNA level were assessed in serum and urine samples using a circulating extracellular RNA detection assay [30].

The EQ-5D-5L questionnaire was used to evaluate QoL across 5 domains (mobility, self-care, usual activities, pain/discomfort, anxiety/depression) [37]; scores for each domain were summarized and an index score was calculated using the United States as the reference country. The EQ-VAS was used to determine the patient’s perception of their overall health on the day of assessment, as rated on a scale of 1 to 100, with higher scores indicating better health [37].

## Data analysis

Formal statistical analyses were not performed, and data were summarized with descriptive statistics. The final analysis included a safety analysis set (ie, all patients who received any amount of the study drug) and a pharmacodynamic (PD) analysis set (ie, all patients who received any amount of the study drug and had ≥ 1 post dose sample for PD analysis). Safety and clinical activity were analyzed in the safety analysis set; PD parameters (urinary ALA and PBG) were analyzed in the PD analysis set.

Porphyria attacks were analyzed according to the total number of events, total person-years (PY; total days/365.25), mean rate (total number of events/total PY) ± standard error of the mean (SEM). Hemin use was summarized for doses administered during the treatment period (on or after the first dose of givosiran); the annualized rate of hemin dosing was summarized in the same manner as the annualized rate of porphyria attacks (ie, mean [SEM] rate, total number of events, and total PY). For analyses of changes from baseline in clinical activity, laboratory, and QoL measurements, baseline values for these parameters were derived in the Phase 1 parent study.

## Results

### Study population

Of the 16 patients enrolled in the Phase 1/2 OLE study, 14 (88%) completed the study (Supplementary material 1). Twelve patients (75%) received givosiran in both the Phase 1 study and the Phase 2 OLE (continuous givosiran), and the remaining 4 patients (25%) received placebo in the Phase 1 study and switched to givosiran during the Phase 2 OLE (placebo-givosiran crossover).

**Fig. 1:**
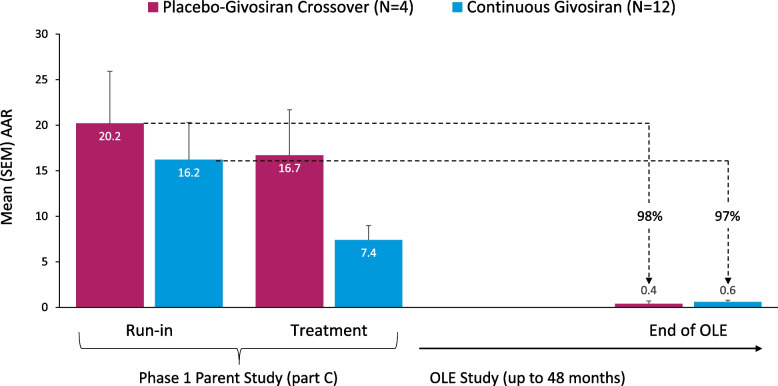
Changes in composite annualized attack rates^a,b^ by study group. AAR, annualized attack rate; IV, intravenous; SEM, standard error of the mean. ^a^Composite attacks requiring hospitalization, urgent healthcare visit, or IV hemin at home. ^b^Data are aggregated across all dose groups, based on an observation time 4.24 PY in the Phase 1 study run-in period and 53.6 PY during the OLE treatment period

Most patients were female (14/16; 88%) and White (13/16; 81%), with a median (range) age of 39.5 (21–60) years (Table 1). At the time of entry into the Phase 1 parent study, patients had a median (range) of 10 porphyria attacks (0–50) in the 12 months before enrollment, and 50% of patients (8/16) were receiving hemin on a scheduled basis; median urinary ALA and PBG levels were 15.8 mmol/mol and 48.0 mmol/mol, respectively (Table 1).

**Table 1:** Baseline demographics and disease characteristics^a^

Statistic	Placebo– Givosiran Crossover (*N* = 4)	Continuous Givosiran (*N* = 12)	Total Givosiran (*N* = 16)
**Age at screening, years, median (range)**	42.0 (27–60)	37.5 (21–59)	39.5 (21–60)
**Female, n (%)**	2 (50)	12 (100)	14 (88)
**Weight, kg, mean (SD)**	91.4 (20.8)	70.7 (15.1)	75.8 (18.5)
**BMI, kg/m**^**2**^**, mean (SD)**	31.1 (4.6)	26.6 (5.8)	27.7 (5.7)
**Race, n (%)**			
White	4 (100)	9 (75)	13 (81)
Black or African American	0	2 (17)	2 (13)
Asian	0	1 (8)	1 (6)
**Ethnicity, n (%)**			
Not Hispanic or Latino	4 (100%)	11 (92)	15 (94)
Not reported	0	1 (8)	1 (6)
**Region, n (%)**			
North America	1 (25)	8 (67)	9 (56)
Europe^b^	3 (75)	4 (33)	7 (44)
**Patients with porphyria attack**^**c**^** in 12 months before enrollment in parent study, n (%)**	4 (100)	11 (92)	15 (94)
Required hospitalization	2 (50)	6 (50)	8 (50)
Treated at outpatient clinic or infusion center	4 (100)	5 (42)	9 (56)
Treated at home	0	5 (42)	5 (31)
**Number of porphyria attacks**^**c**^** in 12 months before enrollment in parent study, median (range)**	10.0 (5–50)	9.5 (0–36)	10.0 (0–50)
**Ever given hemin during an attack before enrollment in parent study, n (%)**	4 (100)	12 (100)	16 (100)
**Taking hemin on scheduled basis just before enrollment in parent study, n (%)**	2 (50)	6 (50)	8 (50)
**Other treatment for porphyria before enrollment in parent study, n (%)**			
Hormone suppression therapy	0	4 (33)	4 (25)
High carbohydrate diet	2 (50)	5 (42)	7 (44)
Glucose infusions	2 (50)	8 (67)	10 (63)
Others	0	4 (33)	4 (25)
**Self-treated at home before enrollment in parent study, n (%)**			
Sugar water	0	2 (17)	2 (13)
High carbohydrates	2 (50)	7 (58)	9 (56)
Opioid analgesic medications	2 (50)	7 (58)	9 (56)
Other	1 (25)	8 (67)	9 (56)
**Urinary ALA, creatinine normalized, mmol/mol**			
N	4	11	15
Median (range)	16.7 (7.5–33.9)	15.4 (1.5–50.5)	15.8 (1.5–50.5)
**Urinary PBG, creatinine normalized, mmol/mol**			
N	4	11	15
Median (range)	46.3 (30.8–51.8)	54.0 (3.2–95.3)	48.0 (3.2–95.3)

*ALA* 5-aminolevulinic acid, *BMI* Body mass index, *PBG* Porphobilinogen, *SD* Standard deviation

^a^Represents safety analysis set. Demographics and characteristics are as recorded in the Phase 1 parent study

^b^Europe includes Sweden and Great Britain

^c^Represents all porphyria attacks, including attacks requiring hospitalization, urgent healthcare visit, or intravenous hemin treatment at home and attacks treated without hemin at home

The median (range) duration of drug exposure was 48.0 (2.1–49.0) months (cumulative exposure, 53.9 PY). Most patients (14/16; 88%) in the Phase 1/2 OLE had received givosiran for ≥ 36 months; and 50% (8/16) had received givosiran for ≥ 48 months. The median (range) number of givosiran doses administered was 43.5 (1–49), with a cumulative total of 623 doses. Across all patients, the total observation time was 4.24 person-years during the Phase 1 run-in period and 53.6 person-years during the OLE study.

### Safety

AEs were reported in all 16 patients (100%), and the majority of AEs were mild or moderate in severity. The most frequently reported AEs were abdominal pain, nasopharyngitis, nausea, fatigue, and injection-site reactions (ISRs) (Table 2). The most common treatment-related AE was ISRs, all of which were of mild or moderate severity and did not lead to treatment discontinuation or study withdrawal. Of the total doses of givosiran administered, 2% (28 of 1246) were associated with ISRs; the most common symptoms included erythema, pruritus, rash, swelling, and discoloration at or near the injection site. One patient had an AE of increased blood homocysteine that was mild in severity, considered possibly related to givosiran, and did not result in any change to givosiran treatment.

**Table 2:** AEs by parent study treatment group

n (%)	Placebo–G﻿ivosiran Crossover (*N* = 4)	Continuous Givosiran (*N* = 12)	Total Givosiran (*N* = 16)
**Any AE**	4 (100)	12 (100)	16 (100)
**AEs occurring in ≥ 25% of patients**			
Abdominal pain	1 (25)	7 (58)	8 (50)
Nasopharyngitis	2 (50)	6 (50)	8 (50)
Nausea	2 (50)	6 (50)	8 (50)
Injection-site reaction^a^	4 (100)	3 (25)	7 (44)
Fatigue	1 (25)	6 (50)	7 (44)
Back pain	2 (50)	3 (25)	5 (31)
Headache	0	5 (42)	5 (31)
Myalgia	2 (50)	3 (25)	5 (31)
Diarrhea	2 (50)	2 (17)	4 (25)
Gastroenteritis	2 (50)	2 (17)	4 (25)
Hypertension	1 (25)	3 (25)	4 (25)
International normalized ratio increased	3 (75)	1 (8)	4 (25)
Lipase increased	1 (25)	3 (25)	4 (25)
Migraine	1 (25)	3 (25)	4 (25)
Oropharyngeal pain	1 (25)	3 (25)	4 (25)
Pain in extremity	2 (50)	2 (17)	4 (25)
Vomiting	1 (25)	3 (25)	4 (25)
**AEs of interest**			
Hepatic AEs^b^	3 (75)	4 (33)	7 (44)
Kidney AEs^c^	1 (25)	4 (33)	5 (31)
Blood homocysteine increased	1 (25)	0	1 (6)
**Any serious AE**	1 (25)	6 (50)	7 (44)
Abdominal pain	0	2 (17)	2 (13)
Anaphylactic reaction	0	1 (8)	1 (6)
*Clostridium difficile* colitis	0	1 (8)	1 (6)
Deep vein thrombosis	1 (25)	0	1 (6)
Dyspnea	0	1 (8)	1 (6)
Forearm fracture	1 (25)	0	1 (6)
Lower limb fracture	1 (25)	0	1 (6)
Mental status changes	0	1 (8)	1 (6)
Pyrexia	0	1 (8)	1 (6)
Respiratory tract infection	0	1 (8)	1 (6)
Sinusitis bacterial	0	1 (8)	1 (6)
Synovitis	0	1 (8)	1 (6)
Tonsillitis	0	1 (8)	1 (6)
**Any severe AE**	3 (75)	4 (33)	7 (44)
**Any AE leading to treatment discontinuation**	0	1 (8)	1 (6)
**Any AE leading to study withdrawal**	0	1 (8)	1 (6)
**Death**	0	0	0

*AE* Adverse event, *ALT* Alanine aminotransferase, *AST* Aspartate aminotransferase, *GFR* Glomerular filtration rate, *GGT* Gamma-glutamyltransferase, *INR* International normalized ratio, *MedDRA* Medical Dictionary for Regulatory Activities, *SAE* Serious adverse event, *SMQ* Standardized MedDRA query

^a^Includes all AEs within the MedDRA high-level term of injection-site reaction

^b^Includes all AEs within SMQ *drug-related hepatic disorders*

^c^Includes all AEs mapping to SMQ *acute renal failure*

Seven patients (44%) experienced serious AEs (Table 2). The only serious AE occurring in > 1 patient was abdominal pain (*n* = 2). One patient with a medical history of allergic asthma and atopy had a serious AE of anaphylaxis considered to be related to treatment. The patient had received 2 doses of givosiran (5 mg/kg 3 months apart) in the Phase 1 study, and the anaphylaxis event occurred 4 months later during her first dose of givosiran (2.5 mg/kg) in the present study. The patient developed urticaria at the injection site extending to her limbs, facial swelling, and hypotension within 3 min of study drug administration; there were no symptoms of airway compromise. This event resolved, and the patient withdrew from the study. Another serious AE of deep venous thrombosis in 1 patient was deemed unrelated to treatment due to the presence of an in-dwelling catheter as a risk for deep vein thrombosis as well as the patient’s known history of chronic hemin use; the event resolved without a change in givosiran dosing.

Seven patients (44%) reported hepatic AEs, most of which were mild or moderate in severity, and all resolved during treatment with givosiran. None of the hepatic AEs were serious, and there were no dose interruptions, changes in dose, or treatment discontinuation. Elevations in liver transaminases were reported in 10 patients (63%). Two patients had transient ALT or aspartate aminotransferase (AST) elevations > 3 and ≤ 5 × ULN without change in total bilirubin. All transaminase elevations resolved with continued givosiran treatment; there were no Hy’s law cases (ie, hepatocellular injury indicated by ALT or AST elevation to ≥ 3 × ULN and increased total bilirubin to ≥ 2 × ULN [38]). Mean values of ALT were generally stable over the course of the study (Supplementary material 2), similar to results of other liver function tests (AST, alkaline phosphatase, bilirubin, and gamma-glutamyltransferase).

**Fig. 2:**
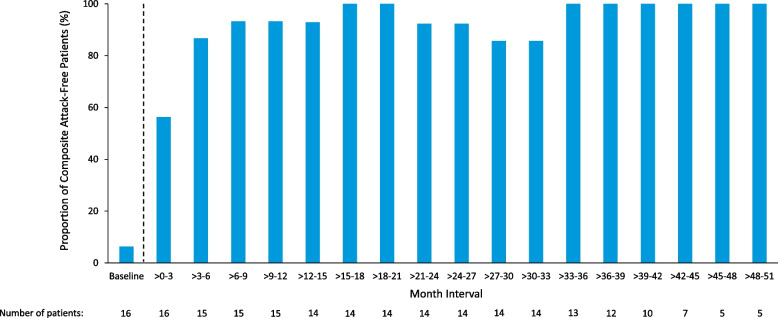
Proportions of composite attack-free patients by 3-month intervals with givosiran 2.5 mg/kg once monthly treatment^a,b^. ^a^Composite attacks included porphyria attacks requiring hospitalization, urgent healthcare visit, or IV hemin administration at home. ^b^The dashed line indicates the gap in time between baseline of the Phase 1 study and the first visit in the OLE study. Baseline is defined as the derived baseline value in the Phase 1 study. Data are based on an observation time of 4.24 person-years in the Phase 1 study run-in period and 53.6 person-years during the OLE treatment period

Kidney AEs were reported in 5 patients (31%); all were mild or moderate in severity. None of the kidney AEs were serious or resulted in treatment interruption or discontinuation. Two patients, both with a long-standing medical history of kidney impairment (eGFR 30–44 mL/min/1.73m^2^ at study entry) and hypertension, had AEs of kidney impairment that were moderate in severity. Mean values for eGFR and creatinine (Cr) were generally stable over the course of the study, with intermittent, small fluctuations observed over time (Supplementary materials 3 and 4).

**Fig. 3:**
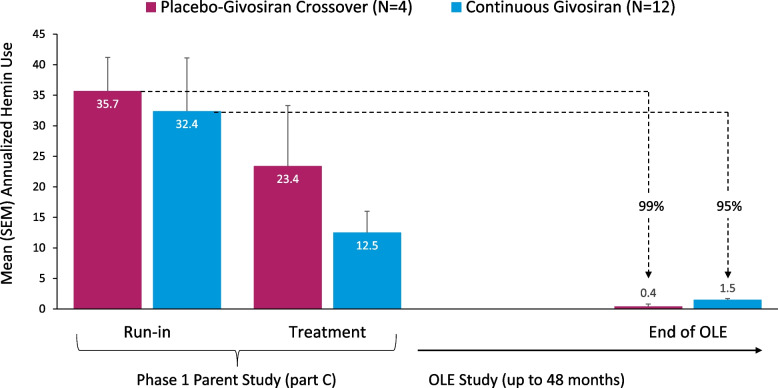
Changes in annualized hemin use^a^ by study group. SEM, standard error of the mean. ^a^Data are aggregated across all dose groups, based on an observation time of 4.24 person-years in the Phase 1 study run-in period and 53.6 person-years during the OLE treatment period

**Fig. 4:**
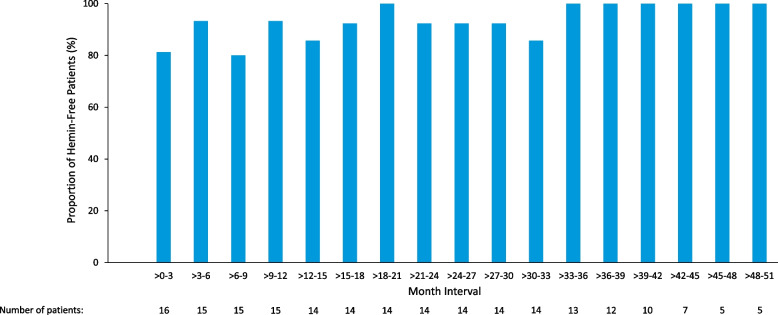
Proportions of hemin-free patients by 3-month intervals with givosiran 2.5 mg/kg once monthly treatment^a^. ^a^Data are aggregated across all dose groups, based on an observation time of 4.24 person-years in the Phase 1 study run-in period and 53.6 person-years during the OLE treatment period

Four patients (25%) had transient increases in lipase levels, with no reported signs or symptoms of pancreatitis. All instances of lipase increase were of moderate severity and resolved during continued treatment with givosiran.

### Clinical activity

The annualized attack rate (AAR) decreased during long-term monthly treatment with givosiran (Fig. 1). In patients originally randomized to placebo during the Phase 1 parent study (placebo-givosiran crossover), the mean (SEM) composite AAR decreased from 20.2 (5.7) during the Phase 1 study run-in period to 0.4 (0.3) during treatment with once-monthly 2.5 mg/kg givosiran in the Phase 1/2 OLE study, indicating a 98% reduction. In patients receiving continuous givosiran therapy during the Phase 1 study and the Phase 1/2 OLE study, mean (SEM) composite AAR decreased from 16.2 (4.1) at the Phase 1 study run-in to 0.6 (0.2) during the Phase 1/2 OLE study, reflecting a 97% reduction. Across all patients in the Phase 1/2 OLE, a 97% reduction in the mean (SEM) composite AAR was observed from the run-in period of the Phase 1 study (17.0 [3.5]) to the once-monthly givosiran 2.5 mg/kg treatment period in the OLE (0.5 [0.2]). The proportion of patients who were attack-free (by 3-month intervals) increased, and this increase was sustained over time; all patients (100%) were attack-free by the Month > 33–36 interval and continued to be attack-free until the end of the study (Fig. 2).

Annualized hemin use substantially decreased during givosiran treatment in both the placebo-givosiran crossover and continuous givosiran treatment groups (Fig. 3). A 99% decrease in mean (SEM) annualized hemin use was observed in the placebo-givosiran crossover group, from 35.7 (5.5) days during the Phase 1 run-in period to 0.4 (0.4) days during givosiran 2.5 mg/kg monthly treatment in the OLE. A 95% reduction in the mean (SEM) annualized hemin use was observed in the continuous givosiran treatment group, from 32.4 (8.7) days during the Phase 1 run-in period to 1.5 (0.9) days/year during the OLE. Across all patients in the Phase 1/2 OLE study, mean (SEM) annualized hemin use decreased from 33.1 (7.0) days during the run-in period in the parent study to 1.2 (0.7) days during treatment with givosiran 2.5 mg/kg once-monthly in the Phase 1/2 OLE, indicating a 96% reduction. Assessment of hemin in 3-month intervals demonstrated that the proportion of patients with 0 days of hemin use increased with time. This increase was sustained, and by Months > 33 to 36, all patients were hemin-free and remained hemin-free until the end of the Phase 1/2 OLE study (Fig. 4).

### Urinary ALA, PBG, and ALAS1 mRNA

Once-monthly treatment with givosiran led to sustained reductions in urinary ALA and PBG levels through Month 48. Median urinary ALA levels decreased from 15.8 mmol/mol Cr at Phase 1 study baseline to 1.0 mmol/mol Cr at OLE Month 48, representing a median reduction of 95% (ULN for ALA, 1.47 mmol/mol Cr) [39]  (Supplementary material 5). Likewise, median urinary PBG levels decreased from 48.0 mmol/mol Cr at Phase 1 study baseline to 1.0 mmol/mol Cr at OLE Month 48, indicating a median reduction of 98% (ULN for PBG, 0.14 mmol/mol Cr) [39] (Supplementary material 6). Circulating hepatic urinary ALAS1 mRNA levels were assessed through OLE Month 18; samples taken during a porphyria attack were excluded from analysis to reduce potential confounding due to hemin administration. Mean urinary ALAS1 mRNA level was 3.51 at baseline of the Phase 1 study, which decreased to 1.54 at OLE Month 12, a mean reduction of 58% (Supplementary material 7). At OLE Month 18, the ALAS mRNA level was 2.09.

**Fig. 5:**
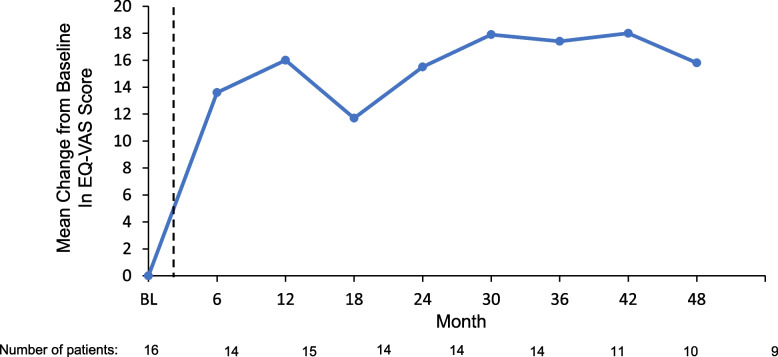
Mean changes in EQ-VAS scores^a^ over time. BL, baseline; EQ-VAS, EuroQol visual analog scale. ^a^The EQ-VAS is a self-rated measure of global health status ranging from 0 (worst imaginable health) to 100 (best imaginable health). Baseline is defined as the derived baseline value in the Phase 1 study. The dotted line indicates the gap in time between baseline of the Phase 1 study and the first visit in the OLE study

**Fig. 6:**
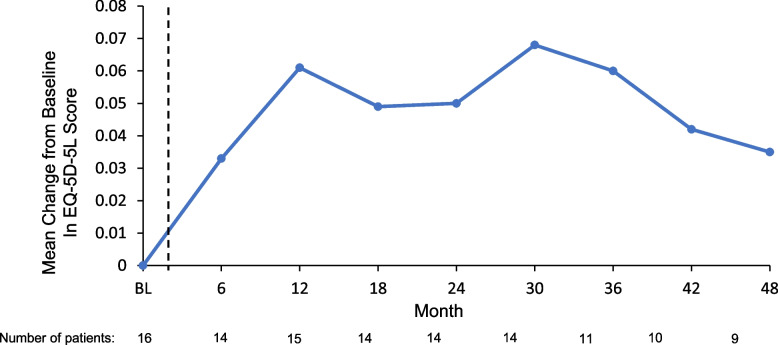
Mean changes in EQ-5D-5L scores^a^ over time. BL, baseline; EQ-5D-5L, Euro Quality of Life Health State Profile Questionnaire. ^a^The EQ-5D-5L summarizes measurements for each of 5 domains (mobility, self-care, usual activities, pain/discomfort, and anxiety/depression). Baseline is defined as the derived baseline value in the Phase 1 study. The dotted line indicates the gap in time between baseline of the Phase 1 study and the first visit in the OLE study

### QoL assessments

The mean (SD) EQ-VAS score increased from 68.9 (20.9) at Phase 1 study baseline to 84.4 (22.4) at OLE Month 48, representing a mean (SD) improvement of 15.8 (13.7) points, indicating a mean improvement of 30% (Fig. 5). The mean increase in EQ-VAS of 15.8 points exceeded the EQ-VAS score range estimated to indicate a minimal clinically important difference (~ 7–10 points) in other chronic disease states. A similar trend was observed in mean (SD) EQ-5D-5L score, which increased from 0.81 (0.11) at Phase 1 study baseline to 0.88 (0.11) at OLE Month 48, representing a mean (SD) improvement of 0.04 (0.09) point (mean improvement of 4.5% from baseline) (Fig. 6). Fewer patients reported difficulty across EQ-5D-5L dimensions of usual activities, pain/discomfort, and anxiety depression at Month 48 (13%, 38%, and 19%, respectively) than at Phase 1 study baseline (69%, 75%, and 63%, respectively).

## Discussion

In this Phase 1/2 OLE study, long-term treatment with once-monthly givosiran 2.5 mg/kg for up to 4 years was well tolerated and had an acceptable safety profile in enrolled patients with AIP. No additional safety concerns emerged during the OLE. Long-term monthly treatment with givosiran led to continuous and sustained reductions in AAR and hemin use over time in patients with AIP. Improvements over time also were observed in QoL measures assessing patient functioning, pain, anxiety, and overall health status. These findings are consistent with the long-term efficacy and safety results of the ENVISION trial evaluating givosiran in patients with AHP and recurrent attacks [33]. Taken together, the long-term clinical trial evidence supports the capacity of givosiran to treat acute disease manifestations in patients with AHP [11, 13, 14, 18, 40, 41].

Most AEs were of mild or moderate severity in the OLE, consistent with previous studies of givosiran [29, 31, 33, 42]. The most commonly reported related AEs in the present study as well as in previous studies of givosiran were ISRs [29, 31, 33, 42]. Two patients discontinued treatment and withdrew from the study: 1 patient due to a serious adverse event of anaphylactic shock, and the other patient due to a lack of marked treatment response. The anaphylaxis reaction occurred in a patient with previous allergic asthma, food allergies, atopic dermatitis, and a prior episode of facial edema after latex contact. This was the sole anaphylaxis event in the study, and corresponding precautions to givosiran labeling were added as a result [23, 25]. All hepatic AEs were transient and resolved with continued givosiran treatment; there were no serious kidney AEs, and most were mild or moderate in severity.

Fluctuations in eGFR and Cr were observed during givosiran treatment during the OLE; generally, the magnitude of these changes was small. Elevated blood homocysteine levels have been reported previously in patients with AHP, including some patients treated with givosiran [42, 43]. In the current study, 1 patient experienced a mild case of elevated homocysteine that did not necessitate a change in givosiran dosing. In an exploratory analysis of AHP clinical trial data, population-level increases in homocysteine with substantial interpatient variation were observed; however, these increases did not correlate with adverse clinical events or changes in the efficacy or safety of givosiran [43]. The long-term consequences of elevated homocysteine levels in patients with AHP remain unknown, and the authors recommended pyridoxine/vitamin B_6_ supplementation [43].

Preclinical and clinical evidence indicates that increased ALAS mRNA levels and consequent accumulation of ALA and PBG cause neurotoxicity and clinical manifestations in patients with AHP, although the exact relationship between elevated ALA/PBG and clinical manifestations is not understood [30, 44, 45]. In the OLE study, ALA and PBG levels showed sustained reductions over the course of long-term givosiran treatment. At baseline in part C of the Phase 1 parent study, circulating hepatic ALAS1 mRNA levels were typically fourfold higher than hepatic ALAS1 mRNA levels in healthy volunteers [30]; ALAS1 mRNA levels were rapidly and stably reduced during treatment with givosiran in the OLE study through the assessment period (Month 18).

Acute porphyria attacks are the most severe and potentially life-threatening manifestations of AHP [9]. Consistent with findings in the ENVISION trial, long-term givosiran therapy led to sustained, substantial decreases in AARs in this OLE study. Overall, once-monthly givosiran 2.5 mg/kg reduced the AAR by 97% relative to the run-in period of the Phase 1 parent study; in the ENVISION trial, the rate of AAR reduction was 92% [33]. Furthermore, all patients (100%) in the OLE became attack-free at Months 33–36 and remained attack-free until the end of the study. In the final 3-month interval of the ENVISION OLE (Months 33–36), > 85% of patients were attack free [33].

IV hemin is indicated for treatment of acute porphyria attacks [46], and is also employed prophylactically to reduce acute attacks, despite accompanying risks of complications such as chronic iron overload, tachyphylaxis, and venous injury [43]. Similar to OLE results for AARs, substantial reductions in hemin use rates were observed with once-monthly givosiran 2.5 mg/kg; overall, hemin use decreased by 97% in the OLE study relative to the run-in period of the Phase 1 study. Parallel to the observed proportions of attack-free patients, 100% of patients in the OLE study were free of hemin use at Months 33–36 and remained hemin-free for the duration of the study. These reductions in hemin use rates and increases in hemin-free proportions were consistent with long-term results in the ENVISION trial [33].

QoL was assessed in the OLE study using the patient-reported EQ-5D-5L, which includes a VAS for rating health [47]. The mean EQ-VAS score in the general US population was computed to be 80.0 (interquartile range, 73–91) on a scale of 0 (worst imaginable health state) to 100 (best imaginable health state) [48]. In the OLE study, the mean EQ-VAS improved from 68.9 points at Phase 1 study baseline, a score indicating impaired quality of life [13, 47], to 84.4 points by the end of long-term givosiran treatment in the OLE study, representing a mean increase of 15.8 points. This increase exceeds the range estimated to indicate a minimally clinically important increase in EQ-VAS score in patients with other chronic diseases, including cancer and chronic obstructive pulmonary disease (approximately 7–10 points) [49, 50]; it should be noted that this threshold has not been validated in individuals with AHP. By OLE Month 48, a marked decrease relative to Phase 1 study baseline was observed in the proportion of patients who reported difficulty in the EQ-5D-5L domains of pain/discomfort, anxiety/depression, and usual activities. Overall, EQ-5D-5L assessments in the OLE study suggest that long-term treatment with givosiran 2.5 mg/kg led to clinically meaningful improvements in QoL, consistent with final results in the ENVISION trial [33]. QoL data from these long-term trials indicate that continued givosiran treatment can reduce chronic symptoms affecting patients’ physical, emotional, social, and financial well-being, disease burden impacts that can be underrecognized due to the relative severity of porphyria attacks.

As expected for a rare disease, the OLE study was limited by the relatively small number of patients relative to other clinical trial populations. Although the open-label nature of the study may have influenced patients’ perceptions regarding changes in QoL experienced during the givosiran treatment period, improvements in clinical parameters (ie, ALA and PBG levels, porphyria attacks, and hemin use) were maintained over the 4-year period.

## Conclusions

This longest follow-up of patients with AIP receiving monthly givosiran therapy (up to 48 months) demonstrated acceptable safety, durable clinical responses, and improvements in QoL assessment scores.

## Supplementary Information

Supplementary Material 1: Figure S1. Phase 1/2 OLE study design and patient disposition. ^a^Patients received givosiran or placebo once monthly (up to 4 doses) or once quarterly during a 12-week period and were followed for an additional 12 weeks after the last injection. ^b^Screening assessment for the OLE was the last assessment performed during the Phase 1 study. If >60 days had elapsed since last Phase 1 study assessment, safety assessments (eg, ECG and clinical laboratory tests) were repeated before administering the first dose of givosiran (OLE Day 1). ^c^Givosiran dosing: patients initially received 2.5 mg/kg once monthly, 5.0 mg/kg once monthly, or 5.0 mg/kg once every 3 months (as per Phase 1 study protocol); all patients transitioned to 2.5 mg/kg once monthly starting August 2017. ^d^Withdrawals were due to treatment-related serious AE of anaphylactic reaction in 1 patient and decision to discontinue treatment due to lack of treatment response in 1 patient. AE, adverse event; ECG, electrocardiogram; OLE, open-label extension.

Supplementary Material 2: Figure S2. Mean (SD) alanine aminotransferase levels (U/L) over time. ALT, alanine aminotransferase; BL, baseline. Baseline is defined as the derived baseline value in the Phase 1 study. The dotted line indicates the gap in time between baseline of the Phase 1 study and the first visit in the OLE study.

Supplementary Material 3: Figure S3. Mean (SD) eGFRs (mL/min/1.73m^2^) over time. BL, baseline; eGFR, estimated glomerular filtration rate; SD, standard deviation. Baseline is defined as the derived baseline value in the Phase 1 study. The dotted line indicates the gap in time between baseline of the Phase 1 study and the first visit in the OLE study.

Supplementary Material 4: Figure S4. Creatinine levels relative to ULN by visit. BL, baseline; ULN, upper limit of normal. Baseline is defined as the derived baseline value in the Phase 1 study. The dotted line indicates the gap in time between baseline of the Phase 1 study and the first visit in the OLE study.

Supplementary Material 5: Figure S5. Median urinary ALA levels^a,b^ (mmol/mol Cr) over time. ALA, 5-aminolevulinic acid; BL, baseline; Cr, creatinine. ULN, upper limit of normal. ^a^Assessed using liquid chromatography-tandem mass spectrometry. ^b^ULN for ALA: 1.47 mmol/mol Cr [39]. Baseline is defined as the derived baseline value in the Phase 1 study. The dotted line indicates the gap in time between baseline of the Phase 1 study and the first visit in the OLE study.

Supplementary Material 6: Figure S6. Median urinary PBG levels^a,b^ (mmol/mol Cr) over time. BL, baseline; Cr, creatinine; PBG, porphobilinogen. ULN, upper limit of normal. ^a^Assessed using liquid chromatography-tandem mass spectrometry. ^b^ULN for PBG: 0.14 mmol/mol Cr [39]. Baseline is defined as the derived baseline value in the Phase 1 study. The dotted line indicates the gap in time between baseline of the Phase 1 study and the first visit in the OLE study.

Supplementary Material 7: Figure S7. Mean (SEM) percent lowering of normalized urinary circulating hepatic ALAS1 mRNA (assessed through Month 18). ALAS1, aminolevulinate synthase 1; BL, baseline; mRNA, messenger RNA. Baseline is defined as the derived baseline value in the Phase 1 study; the dashed line indicates the gap in time between baseline of the Phase 1 study and the first visit in the OLE study.

## Data Availability

De-identified individual participant data that support these results will be made available in a secure-access environment 12 months after study completion. Access will be provided contingent upon the approval of a research proposal and the execution of a data sharing agreement.

## Abbreviations

AAR: Annualized attack rate
AE: Adverse event
AHP: Acute hepatic porphyria
AIP: Acute intermittent porphyria
ALA: 5-Aminolevulinic acid
ALAS1: Aminolevulinate synthase 1
ALT: Alanine aminotransferase
AST: Aspartate aminotransferase
BL: Baseline
Cr: Creatinine
eGFR: Estimated glomerular filtration rate
EQ-5D-5L: Euro Quality of Life Health State Profile Questionnaire
EQ-VAS: EuroQoL visual analogue scale
HCP: Hereditary coproporphyria
HMBS: Hydroxymethylbilane synthase
ISR: Injection-site reaction
IV: Intravenous
mRNA: Messenger RNA
OLE: Open-label extension
PBG: Porphobilinogen
PD: Pharmacodynamics
PY: Patient-year(s)
QoL: Quality of life
RNA: Ribonucleic acid
SD: Standard deviation
SEM: Standard error of the mean
ULN: Upper limit of normal
VP: Variegate porphyria

## References

[1] Anderson KE, Lobo R, Salazar D, Schloetter M, Spitzer G, White AL, et al. Biochemical diagnosis of acute hepatic porphyria: updated expert recommendations for primary care physicians. Am J Med Sci. 2021;362(2):113–21. 33865828 10.1016/j.amjms.2021.03.004

[2] Puy H, Gouya L, Deybach JC. Porphyrias. Lancet. 2010;375(9718):924–37. 20226990 10.1016/S0140-6736(09)61925-5

[3] Wang B, Rudnick S, Cengia B, Bonkovsky HL. Acute hepatic porphyrias: review and recent progress. Hepatol Commun. 2019;3(2):193–206. 30766957 10.1002/hep4.1297 PMC6357830

[4] Anderson KE, Bloomer JR, Bonkovsky HL, Kushner JP, Pierach CA, Pimstone NR, et al. Recommendations for the diagnosis and treatment of the acute porphyrias. Ann Intern Med. 2005;142(6):439–50. 15767622 10.7326/0003-4819-142-6-200503150-00010

[5] Balwani M, Wang B, Anderson KE, Bloomer JR, Bissell DM, Bonkovsky HL, et al. Acute hepatic porphyrias: recommendations for evaluation and long-term management. Hepatology. 2017;66:1314–22. 28605040 10.1002/hep.29313 PMC5605422

[6] Bissell DM, Anderson KE, Bonkovsky HL. Porphyria. N Engl J Med. 2017;377(9):862–72. 28854095 10.1056/NEJMra1608634

[7] Ramanujam VM, Anderson KE. Porphyria diagnostics-part 1: a brief overview of the porphyrias. Curr Protoc Hum Genet. 2015;86:17.20.1–17.20.6. 10.1002/0471142905.hg1720s86 PMC4640448 26132003

[8] Wheeden K, Lyon Howe D, Burrell S, Gill L, Chamberlayne J, Williams ER, et al. Patient perspective on acute hepatic porphyria with sporadic attacks: a chronic disease with substantial health-related quality of life impacts. Adv Ther. 2022;39(9):4330–5. 35907153 10.1007/s12325-022-02172-8 PMC9402748

[9] Stein PE, Edel Y, Mansour R, Mustafa RA, Sandberg S. Key terms and definitions in acute porphyrias: Results of an international Delphi consensus led by the European porphyria network. J Inherit Metab Dis. 2023;46(4):662–74. 37067064 10.1002/jimd.12612

[10] Bonkovsky HL, Maddukuri VC, Yazici C, Anderson KE, Bissell DM, Bloomer JR, et al. Acute porphyrias in the USA: features of 108 subjects from porphyrias consortium. Am J Med. 2014;127(12):1233–41. 25016127 10.1016/j.amjmed.2014.06.036 PMC4563803

[11] Buendía-Martínez J, Barreda-Sánchez M, Rodríguez-Peña L, Ballesta-Martínez MJ, López-González V, Sánchez-Soler MJ, et al. Health impact of acute intermittent porphyria in latent and non-recurrent attacks patients. Orphanet J Rare Dis. 2021;16(1):106. 33639982 10.1186/s13023-021-01742-3 PMC7913433

[12] Dickey A, Wheeden K, Burrell S, Falchetto R, Barman-Aksozen J, Bulkley A, et al. Impact of acute hepatic porphyria attack frequency on patient-reported outcomes: results from the Porphyria Worldwide Patient Experience Research (POWER) study [abstract FRI251]. J Hepatol. 2022;77(suppl 1):S516. 10.1002/jmd2.12343 PMC9830021 36636593

[13] Gouya L, Ventura P, Balwani M, Bissell DM, Rees DC, Stolzel U, et al. EXPLORE: a prospective, multinational, natural history study of patients with acute hepatic porphyria with recurrent attacks. Hepatology. 2020;71(5):1546–58. 31512765 10.1002/hep.30936 PMC7255459

[14] Naik H, Stoecker M, Sanderson SC, Balwani M, Desnick RJ. Experiences and concerns of patients with recurrent attacks of acute hepatic porphyria: a qualitative study. Mol Genet Metab. 2016;119(3):278–83. 27595545 10.1016/j.ymgme.2016.08.006 PMC5083146

[15] Stein PE, Badminton MN, Barth JH, Rees DC, Sarkany R, Stewart MF, et al. Acute intermittent porphyria: fatal complications of treatment. Clin Med (London). 2012;12(3):293–4. 22783787 10.7861/clinmedicine.12-3-293 PMC4953498

[16] Pischik E, Kauppinen R. An update of clinical management of acute intermittent porphyria. Appl Clin Genet. 2015;8:201–14. 26366103 10.2147/TACG.S48605 PMC4562648

[17] Wang B. The acute hepatic porphyrias. Transl Gastroenterol Hepatol. 2021;6:24. 33824928 10.21037/tgh-2020-01 PMC7838531

[18] Stein PE, Badminton MN, Rees DC. Update review of the acute porphyrias. Br J Haematol. 2017;176(4):527–38. 27982422 10.1111/bjh.14459

[19] Harper P, Sardh E. Management of acute intermittent porphyria. Expert Opin Orphan Drugs. 2014;2(4):349–68.

[20] Marsden JT, Guppy S, Stein P, Cox TM, Badminton M, Gardiner T, et al. Audit of the use of regular haem arginate infusions in patients with acute porphyria to prevent recurrent symptoms. JIMD Rep. 2015;22:57–65. 25762493 10.1007/8904_2015_411 PMC4486272

[21] Bonkovsky HL, Dixon N, Rudnick S. Pathogenesis and clinical features of the acute hepatic porphyrias (AHPs). Mol Genet Metab. 2019;128(3):213–8. 30987916 10.1016/j.ymgme.2019.03.002 PMC6754303

[22] Anderson KE. Acute hepatic porphyrias: current diagnosis & management. Mol Genet Metab. 2019;128(3):219–27. 31311713 10.1016/j.ymgme.2019.07.002 PMC6911835

[23] Givlaari [package insert]. Cambridge, MA, USA: Alnylam Pharmaceuticals; 2023 February 2023.

[24] Alnylam announces approval of GIVLAARI® (givosiran) in Brazil for the treatment of acute hepatic porphyria (AHP) in adults [press release] Sao Paulo, Brazil: Alnylam Pharmaceuticals; 2020 [July 20, 2020]. Available from: https://investors.alnylam.com/sites/default/files/GIVLAARI-Brazil-Approval-Press-Release.pdf.

[25] Givlaari [summary of product characteristics] Amsterdam, The Netherlands: Alnylam Netherlands; 2021 [January 1, 2021]. Available from: https://www.ema.europa.eu/en/documents/product-information/givlaari-epar-product-information_en.pdf.

[26] Givlaari Canada [product monograph]. Amsterdam, Netherlands: Alnylam Netherlands; 2020 October 2020.

[27] Givlaari, solution for injection (Givosiranum) Switzerland: swissmedic; 2021 [March 29, 2021]. Available from: https://www.swissmedic.ch/swissmedic/en/home/humanarzneimittel/authorisations/new-medicines/givlaari-injektionsloesung-givosiranum.html.

[28] Obtained manufacturing and marketing approval for "Giblari" for the treatment of acute hepatic porphyria [press release] Tokyo, Japan: Alnylam Japan Co., Ltd.; 2021 [June 23, 2021]. Available from: https://www.alnylam.jp/sites/default/files/news-articles/Japan_Givo_Approval_Press_Release_0.pdf.

[29] Balwani M, Sardh E, Ventura P, Peiró PA, Rees DC, Stölzel U, et al. Phase 3 trial of RNAi therapeutic givosiran for acute intermittent porphyria. N Engl J Med. 2020;382(24):2289–301. 32521132 10.1056/NEJMoa1913147

[30] Chan A, Liebow A, Yasuda M, Gan L, Racie T, Maier M, et al. Preclinical development of a subcutaneous *ALAS1* RNAi therapeutic for treatment of hepatic porphyrias using circulating RNA quantification. Mol Ther Nucleic Acids. 2015;4: e263. 26528940 10.1038/mtna.2015.36 PMC4877445

[31] Sardh E, Harper P, Balwani M, Stein P, Rees D, Bissell DM, et al. Phase 1 trial of an RNA interference therapy for acute intermittent porphyria. N Engl J Med. 2019;380(6):549–58. 30726693 10.1056/NEJMoa1807838

[32] Vassiliou D, Sardh E, Harper P, Najafian N, Simon A, Burke A, et al., editors. A drug-drug interaction study to investigate the effect of givosiran on the activity of 5 major drug metabolizing CYP450 enzymes in subjects with acute intermittent porphyria (AIP) who are chronic high excreters (CHE). International Congress on Porphyrins and Porphyrias; 2019 September 10, 2019; Milan, Italy.

[33] Kuter DJ, Bonkovsky HL, Monroy S, Ross G, Guillén-Navarro E, Cappellini MD, et al. Efficacy and safety of givosiran for acute hepatic porphyria: Final results of the randomized phase III ENVISION trial. J Hepatol. 2023;79(5):1150–8. 37479139 10.1016/j.jhep.2023.06.013

[34] World Medical Association. WMA Declaration of Helsinki - ethical principles for medical research involving human subjects Ferney-Voltaire, France: World Medical Association; 2013 [Available from: https://www.wma.net/policies-post/wma-declaration-of-helsinki-ethical-principles-for-medical-research-involving-human-subjects/. 10.1001/jama.2013.281053 24141714

[35] International Council for Harmonisation Working Group. ICH harmonised tripartite guideline: guideline for good clinical practice E6(R1): International Conference on Harmonisation of Technical Requirements for Registration of Pharmaceuticals for Human Use; 1996 [June 10, 1996]. Available from: https://www.ich.org/page/efficacy-guidelines.

[36] The Declaration of Istanbul on Organ Trafficking and Transplant Tourism: The Transplantation Society; 2018. Available from: https://www.declarationofistanbul.org/images/documents/doi_2018_English.pdf. 10.1097/TP.0b013e318185ffc9 18946336

[37] EuroQol Research Foundation. EQ-5D-5L User Guide. Rotterdam, Netherlands: EuroQol Research Foundation; 2019.

[38] Temple R. Hy’s law: predicting serious hepatotoxicity. Pharmacoepidemiol Drug Saf. 2006;15(4):241–3. 16552790 10.1002/pds.1211

[39] Agarwal S, Habtemarium B, Xu Y, Simon AR, Kim JB, Robbie GJ. Normal reference ranges for urinary δ-aminolevulinic acid and porphobilinogen levels. JIMD Rep. 2021;57(1):85–93. 33473344 10.1002/jmd2.12173 PMC7802627

[40] Simon A, Pompilus F, Querbes W, Wei A, Strzok S, Penz C, et al. Patient perspective on acute intermittent porphyria with frequent attacks: a disease with intermittent and chronic manifestations. Patient. 2018;11(5):527–37. 29915990 10.1007/s40271-018-0319-3 PMC6132435

[41] Dickey A, Wheeden K, Lyon D, Burrell S, Hegarty S, Falchetto R, et al. Quantifying the impact of symptomatic acute hepatic porphyria on well-being via patient-reported outcomes: results from the Porphyria Worldwide Patient Experience Research (POWER) study. JIMD Rep. 2023;64(1):104–13. 36636593 10.1002/jmd2.12343 PMC9830021

[42] Ventura P, Bonkovsky HL, Gouya L, Aguilera-Peiró P, Bissell DM, Stein PE, et al. Efficacy and safety of givosiran for acute hepatic porphyria: 24-month interim analysis of the randomized phase 3 ENVISION study. Liver Int. 2022;42(1):161–72. 34717041 10.1111/liv.15090 PMC9299194

[43] Ventura P, Sardh E, Longo N, Balwani M, Plutzky J, Gouya L, et al. Hyperhomocysteinemia in acute hepatic porphyria (AHP) and implications for treatment with givosiran. Expert Rev Gastroenterol Hepatol. 2022;16(9):879–94. 35929959 10.1080/17474124.2022.2110469

[44] Bissell DM, Wang B. Acute hepatic porphyria. J Clin Transl Hepatol. 2015;3(1):17–26. 26357631 10.14218/JCTH.2014.00039 PMC4542079

[45] Marsden JT, Rees DC. Urinary excretion of porphyrins, porphobilinogen and δ-aminolaevulinic acid following an attack of acute intermittent porphyria. J Clin Pathol. 2014;67(1):60–5. 23908454 10.1136/jclinpath-2012-201367

[46] Panhematin [package insert]. Lebanon, NJ: Recordati Rare Diseases Inc.; 2017.

[47] Herdman M, Gudex C, Lloyd A, Janssen M, Kind P, Parkin D, et al. Development and preliminary testing of the new five-level version of EQ-5D (EQ-5D-5L). Qual Life Res. 2011;20(10):1727–36. 21479777 10.1007/s11136-011-9903-x PMC3220807

[48] Szende A, Janssen B, Cabases J, editors. Self-Reported Population Health: An International Perspective Based on EQ-5D. Dordrecht, Netherlands: Springer; 2014. 29787044

[49] Zanini A, Aiello M, Adamo D, Casale S, Cherubino F, Della Patrona S, et al. Estimation of minimal clinically important difference in EQ-5D visual analog scale score after pulmonary rehabilitation in subjects with COPD. Respir Care. 2015;60(1):88–95. 25336531 10.4187/respcare.03272

[50] Pickard AS, Neary MP, Cella D. Estimation of minimally important differences in EQ-5D utility and VAS scores in cancer. Health Qual Life Outcomes. 2007;5:70. 18154669 10.1186/1477-7525-5-70 PMC2248572

