# Supplementary material for: Long-term follow-up of givosiran treatment in patients with acute intermittent porphyria from a phase 1/2, 48-month open-label extension study
Source: Orphanet J Rare Dis. 2024 Oct 3;19:365. doi: 10.1186/s13023-024-03284-w (PMC11448181; doi:10.1186/s13023-024-03284-w)

Additional file 1. Phase 1/2 OLE study design and patient disposition

[a]Patients received givosiran or placebo once monthly (up to 4 doses) or once quarterly (up to 2 doses) during a 12-week period and were followed for an additional 12 weeks after the last injection.

[b]Screening assessment for the OLE was the last assessment performed during the Phase 1 study. If >60 days had elapsed since last Phase 1 study assessment, safety assessments (eg, ECG and clinical laboratory tests) were repeated before administering the first dose of givosiran (OLE Day 1).

[c]Givosiran dosing: patients initially received 2.5 mg/kg once monthly, 5.0 mg/kg once monthly, or 5.0 mg/kg once every 3 months (as per Phase 1 study protocol); all patients transitioned to 2.5 mg/kg once monthly starting August 2017.

[d]Withdrawals were due to treatment-related serious AE of anaphylactic reaction in 1 patient and decision to discontinue treatment due to lack of treatment response in 1 patient.

AE, adverse event; ECG, electrocardiogram; OLE, open-label extension.

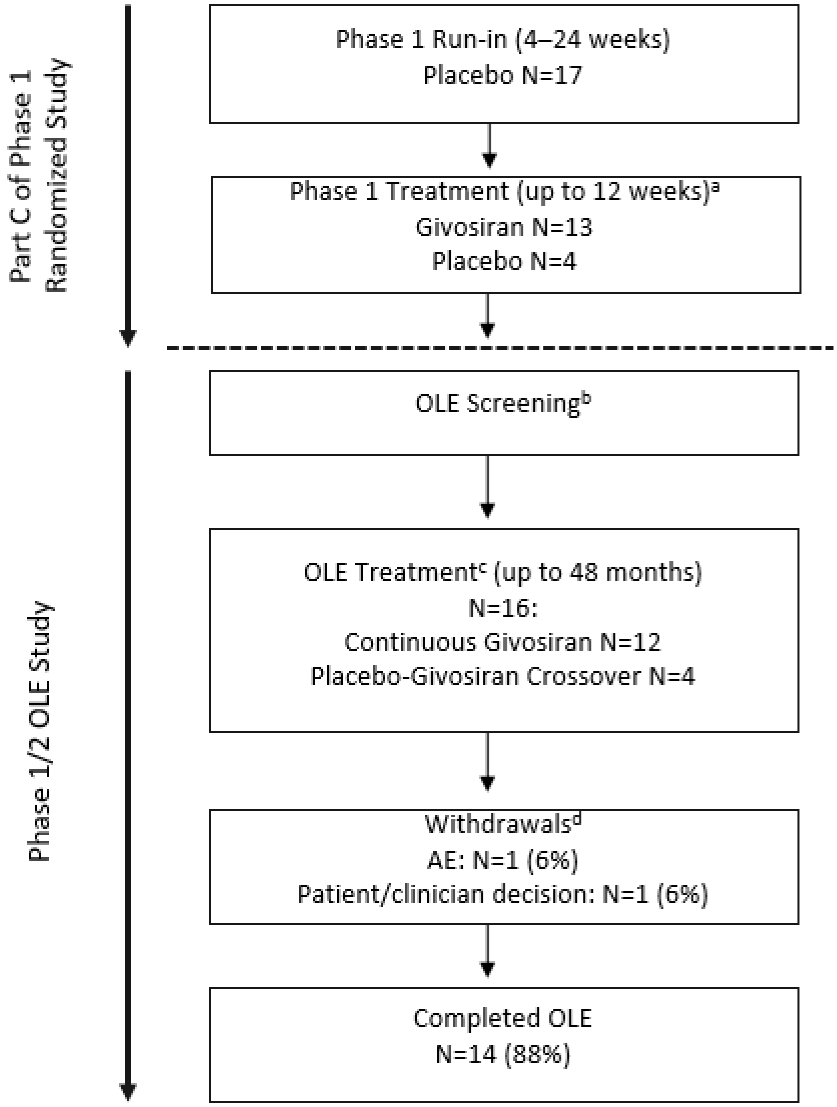

Supplement: Supplementary file 1 — Supplementary Material 1: Figure S1. Phase 1/2 OLE study design and patient disposition. aPatients received givosiran or placebo once monthly (up to 4 doses) or once quarterly during a 12-week period and were followed for an additional 12 weeks after the last injection. bScreening assessment for the OLE was the last assessment performed during the Phase 1 study. If >60 days had elapsed since last Phase 1 study assessment, safety assessments (eg, ECG and clinical laboratory tests) were repeated before administering the first dose of givosiran (OLE Day 1). cGivosiran dosing: patients initially received 2.5 mg/kg once monthly, 5.0 mg/kg once monthly, or 5.0 mg/kg once every 3 months (as per Phase 1 study protocol); all patients transitioned to 2.5 mg/kg once monthly starting August 2017. dWithdrawals were due to treatment-related serious AE of anaphylactic reaction in 1 patient and decision to discontinue treatment due to lack of treatment response in 1 patient. AE, adverse event; ECG, electrocardiogram; OLE, open-label extension. [file 13023_2024_3284_MOESM1_ESM.pdf]
