# Supplementary material for: Long-term follow-up of givosiran treatment in patients with acute intermittent porphyria from a phase 1/2, 48-month open-label extension study
Source: Orphanet J Rare Dis. 2024 Oct 3;19:365. doi: 10.1186/s13023-024-03284-w (PMC11448181; doi:10.1186/s13023-024-03284-w)
Supplement: Supplementary file 2 — Supplementary Material 2: Figure S2. Mean (SD) alanine aminotransferase levels (U/L) over time. ALT, alanine aminotransferase; BL, baseline. Baseline is defined as the derived baseline value in the Phase 1 study. The dotted line indicates the gap in time between baseline of the Phase 1 study and the first visit in the OLE study. [file 13023_2024_3284_MOESM2_ESM.pdf]

Additional file 2. Mean (SD) alanine aminotransferase levels (U/L) over time

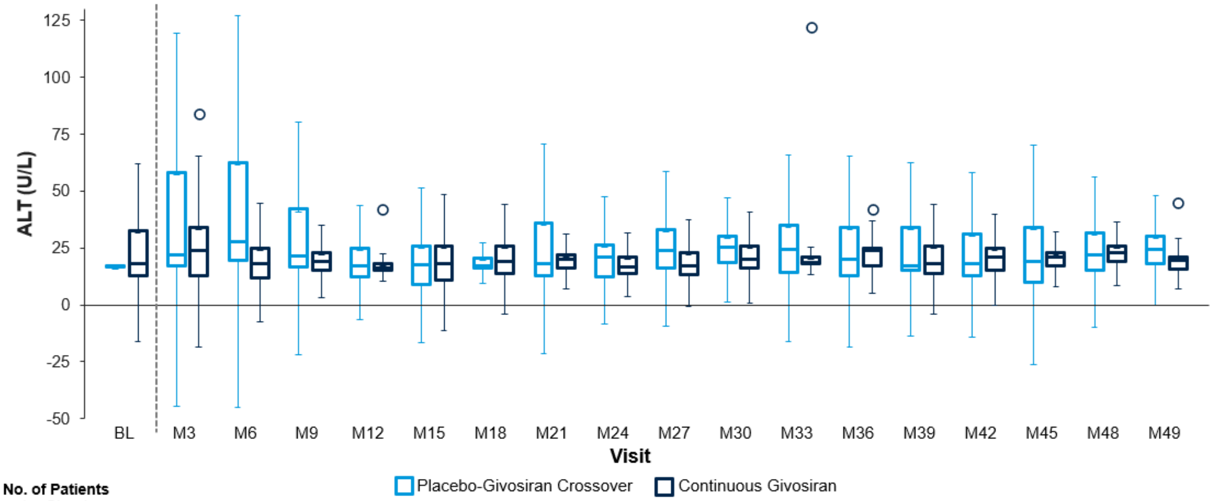

No. of Patients	BL	M3	M6	M9	M12	M15	M18	M21	M24	M27	M30	M33	M36	M39	M42	M45	M48	M49
Placebo-Givosiran Crossover	4	4	4	4	4	2	4	3	4	4	4	4	3	3	3	3	4	4
Continuous Givosiran	12	11	11	11	11	11	10	9	10	8	10	10	9	7	7	5	6	8

ALT, alanine aminotransferase; BL, baseline.

Baseline is defined as the derived baseline value in the Phase 1 study. The dotted line indicates the gap in time between baseline of the Phase 1 study and the first visit in the OLE study.
