# Supplementary material for: Long-term follow-up of givosiran treatment in patients with acute intermittent porphyria from a phase 1/2, 48-month open-label extension study
Source: Orphanet J Rare Dis. 2024 Oct 3;19:365. doi: 10.1186/s13023-024-03284-w (PMC11448181; doi:10.1186/s13023-024-03284-w)
Supplement: Supplementary file 4 — Supplementary Material 4: Figure S4. Creatinine levels relative to ULN by visit. BL, baseline; ULN, upper limit of normal. Baseline is defined as the derived baseline value in the Phase 1 study. The dotted line indicates the gap in time between baseline of the Phase 1 study and the first visit in the OLE study. [file 13023_2024_3284_MOESM4_ESM.pdf]

Additional file 4. Creatinine levels relative to ULN by visit

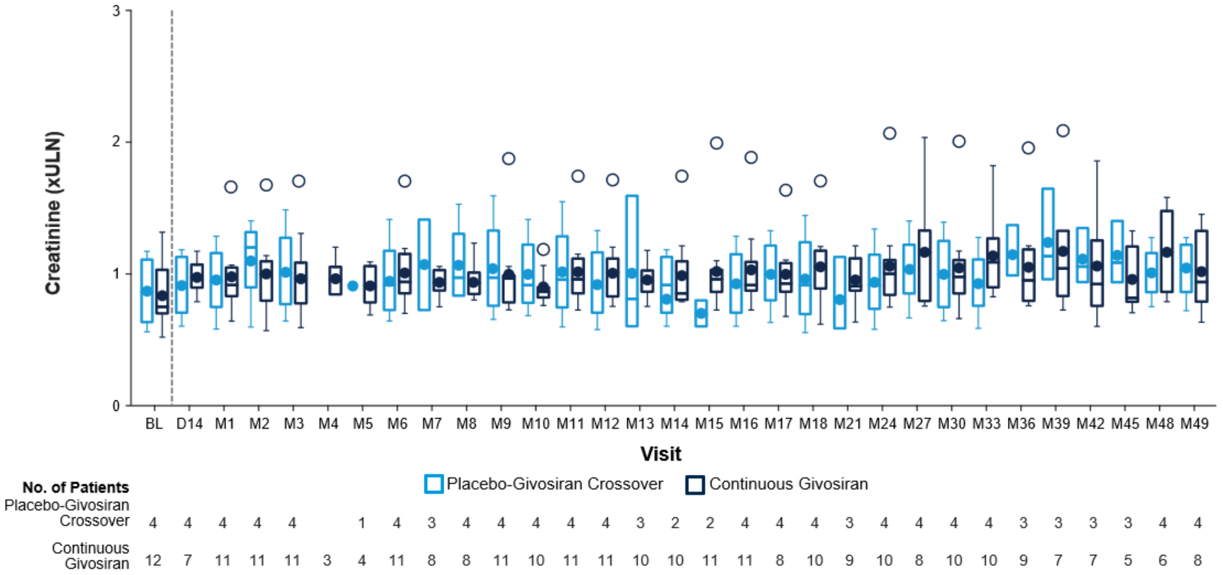

BL, baseline; ULN, upper limit of normal.

Baseline is defined as the derived baseline value in the Phase 1 study. The dotted line indicates the gap in time between baseline of the Phase 1 study and the first visit in the OLE study.
