# Supplementary material for: Long-term follow-up of givosiran treatment in patients with acute intermittent porphyria from a phase 1/2, 48-month open-label extension study
Source: Orphanet J Rare Dis. 2024 Oct 3;19:365. doi: 10.1186/s13023-024-03284-w (PMC11448181; doi:10.1186/s13023-024-03284-w)
Supplement: Supplementary file 5 — Supplementary Material 5: Figure S5. Median urinary ALA levelsa,b (mmol/mol Cr) over time. ALA, 5-aminolevulinic acid; BL, baseline; Cr, creatinine. ULN, upper limit of normal. aAssessed using liquid chromatography-tandem mass spectrometry. bULN for ALA: 1.47 mmol/mol Cr [39]. Baseline is defined as the derived baseline value in the Phase 1 study. The dotted line indicates the gap in time between baseline of the Phase 1 study and the first visit in the OLE study. [file 13023_2024_3284_MOESM5_ESM.pdf]

**Additional file 5.** Median urinary ALA levels[a,b] (mmol/mol Cr) over time

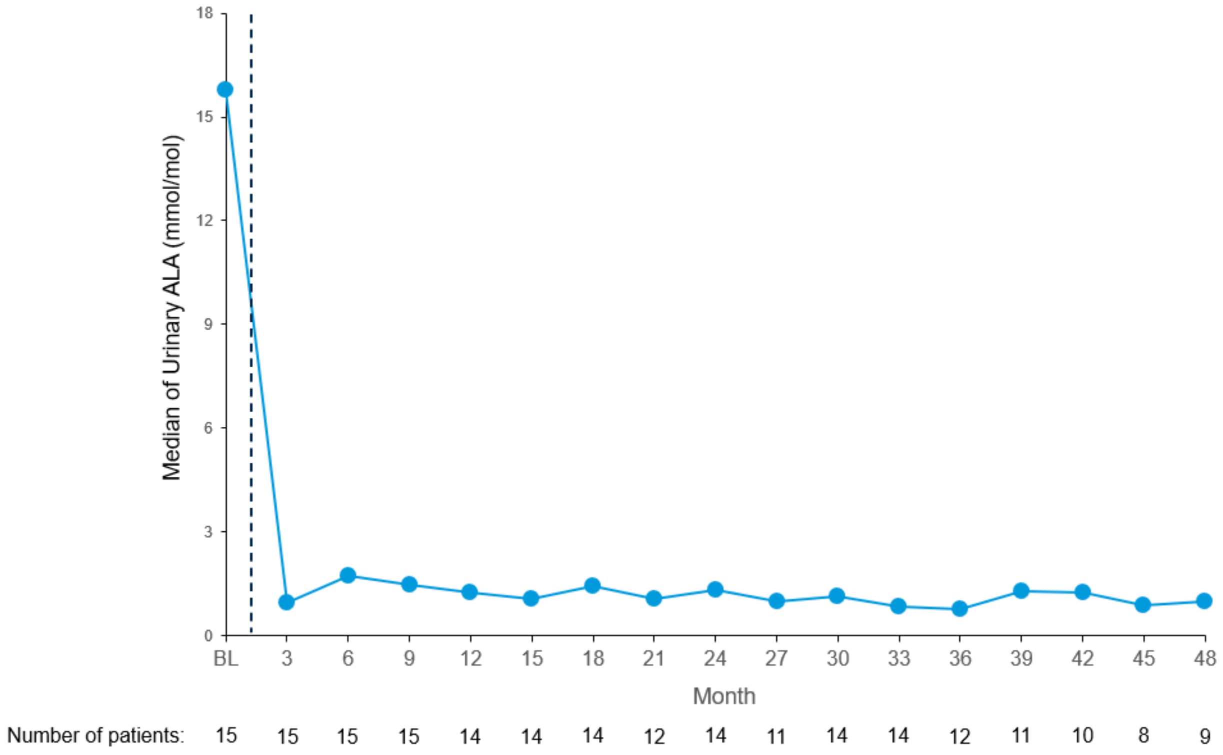


ALA, 5-aminolevulinic acid; BL, baseline; Cr, creatinine. ULN, upper limit of normal.
[a]Assessed using liquid chromatography-tandem mass spectrometry.
[b]ULN for ALA: 1.47 mmol/mol Cr.[1]
Baseline is defined as the derived baseline value in the Phase 1 study. The dotted line indicates the gap in time between baseline of the Phase 1 study and the first visit in the OLE study.

## Reference

1. Agarwal S, Habtemarium B, Xu Y, Simon AR, Kim JB, Robbie GJ. Normal reference ranges for urinary δ-aminolevulinic acid and porphobilinogen levels. JIMD Rep. 2021;57:85-93.
