# Supplementary material for: Long-term follow-up of givosiran treatment in patients with acute intermittent porphyria from a phase 1/2, 48-month open-label extension study
Source: Orphanet J Rare Dis. 2024 Oct 3;19:365. doi: 10.1186/s13023-024-03284-w (PMC11448181; doi:10.1186/s13023-024-03284-w)
Supplement: Supplementary file 6 — Supplementary Material 6: Figure S6. Median urinary PBG levelsa,b (mmol/mol Cr) over time. BL, baseline; Cr, creatinine; PBG, porphobilinogen. ULN, upper limit of normal. aAssessed using liquid chromatography-tandem mass spectrometry. bULN for PBG: 0.14 mmol/mol Cr [39]. Baseline is defined as the derived baseline value in the Phase 1 study. The dotted line indicates the gap in time between baseline of the Phase 1 study and the first visit in the OLE study. [file 13023_2024_3284_MOESM6_ESM.pdf]

Additional file 6. Median urinary PBG levels[a,b] (mmol/mol Cr) over time

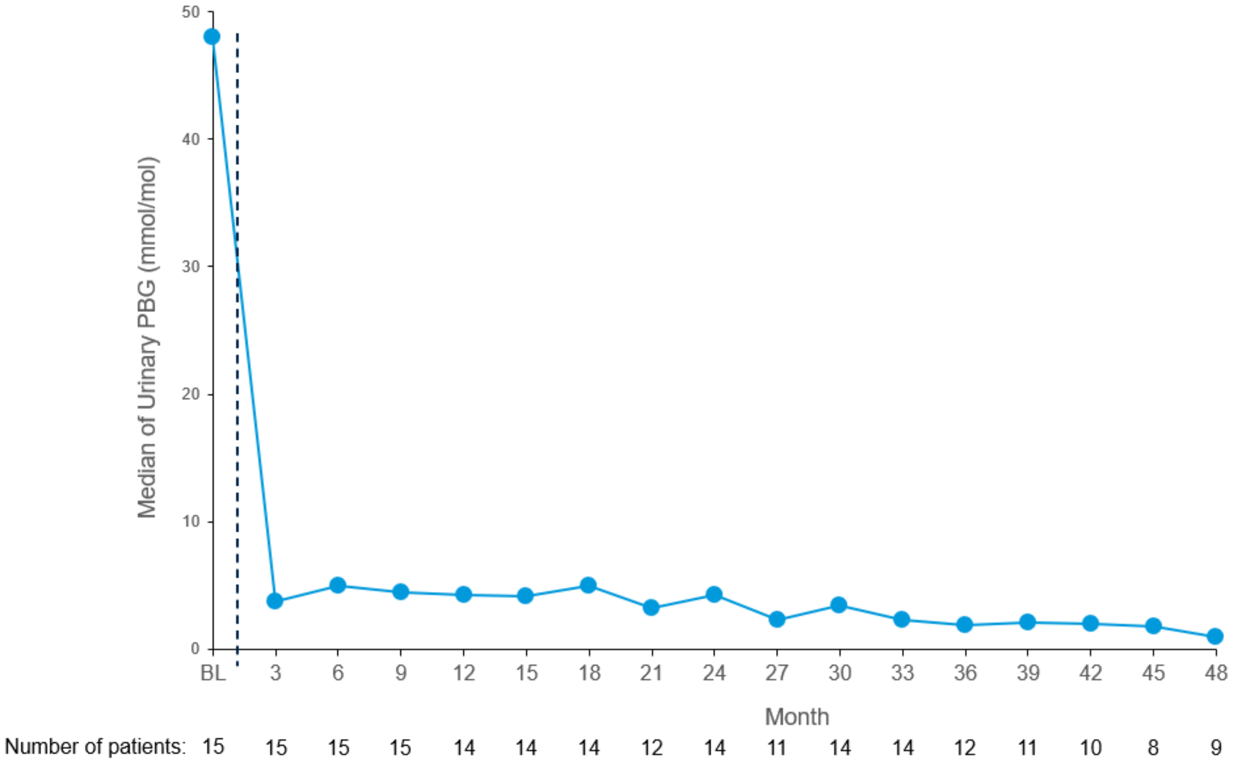

BL, baseline; Cr, creatinine; PBG, porphobilinogen. ULN, upper limit of normal.

[a]Assessed using liquid chromatography-tandem mass spectrometry.

[b]ULN for PBG: 0.14 mmol/mol Cr.[1]
Baseline is defined as the derived baseline value in the Phase 1 study. The dotted line indicates the gap in time between baseline of the Phase 1 study and the first visit in the OLE study.

Reference

1. Agarwal S, Habtemarium B, Xu Y, Simon AR, Kim JB, Robbie GJ. Normal reference ranges for urinary δ-aminolevulinic acid and porphobilinogen levels. JIMD Rep. 2021;57:85-93.
