# Supplementary material for: Long-term follow-up of givosiran treatment in patients with acute intermittent porphyria from a phase 1/2, 48-month open-label extension study
Source: Orphanet J Rare Dis. 2024 Oct 3;19:365. doi: 10.1186/s13023-024-03284-w (PMC11448181; doi:10.1186/s13023-024-03284-w)
Supplement: Supplementary file 7 — Supplementary Material 7: Figure S7. Mean (SEM) percent lowering of normalized urinary circulating hepatic ALAS1 mRNA (assessed through Month 18). ALAS1, aminolevulinate synthase 1; BL, baseline; mRNA, messenger RNA. Baseline is defined as the derived baseline value in the Phase 1 study; the dashed line indicates the gap in time between baseline of the Phase 1 study and the first visit in the OLE study. [file 13023_2024_3284_MOESM7_ESM.pdf]

Additional file 7. Mean (SEM) percent lowering of normalized urinary circulating hepatic ALAS1 mRNA (assessed through Month 18)

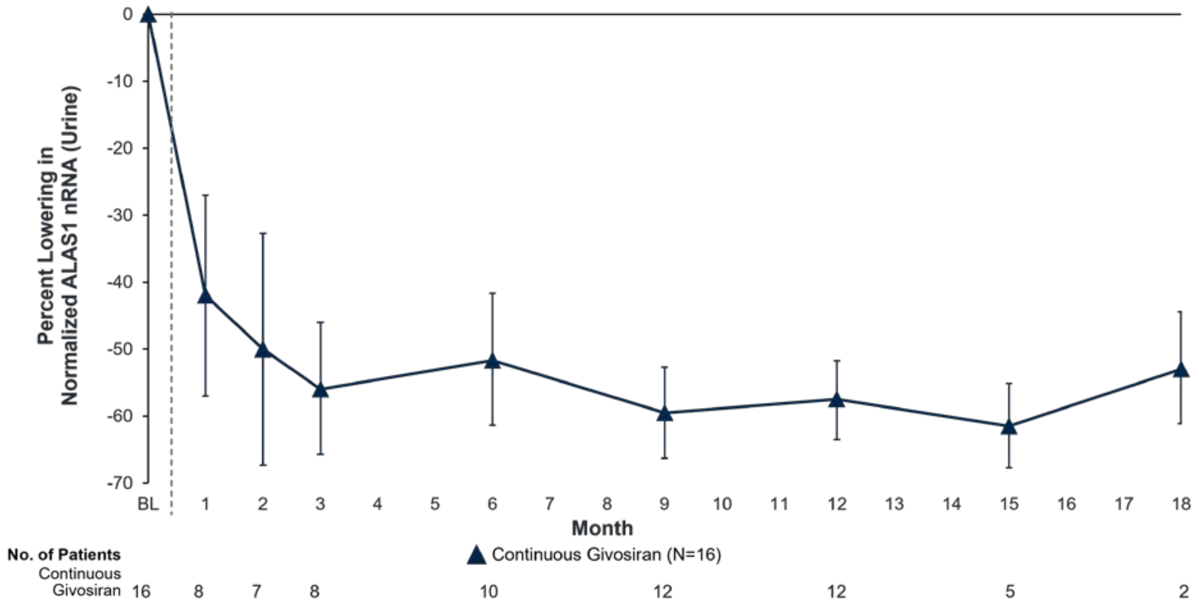

ALAS1, aminolevulinate synthase 1; BL, baseline; mRNA, messenger RNA.

Baseline is defined as the derived baseline value in the Phase 1 study; the dashed line indicates the gap in time between baseline of the Phase 1 study and the first visit in the OLE study.
